# Positive Predictive Value of MOG-IgG for Clinically Defined MOG-AD Within a Real-World Cohort

**DOI:** 10.3389/fneur.2022.947630

**Published:** 2022-06-20

**Authors:** Giovanna S. Manzano, Rebecca Salky, Farrah J. Mateen, Eric C. Klawiter, Tanuja Chitnis, Michael Levy, Marcelo Matiello

**Affiliations:** ^1^Department of Neurology, Massachusetts General Hospital, Harvard Medical School, Boston, MA, United States; ^2^Department of Neurology, Brigham and Women's Hospital, Harvard Medical School, Boston, MA, United States

**Keywords:** myelin oligodendrocyte glycoprotein (MOG), MOG-AD, MOG-IgG, positive predictive value (PPV), antibody testing

## Abstract

Myelin oligodendrocyte glycoprotein antibody associated disease (MOG-AD) is a CNS demyelinating disease, typically presenting with optic neuritis, transverse myelitis, and/or ADEM-like syndromes. The positive predictive value (PPV) of MOG-IgG testing by live cell-based assay was reported to be 72% in a study performed at the Mayo Clinic using a cut-off of 1:20. PPV may vary depending upon the tested population, thus supporting further investigation of MOG-IgG testing at other centers. In this real-world institutional cohort study, we determined the PPV of serum MOG-IgG for clinically defined MOG-AD in our patient population. The Massachusetts General Brigham Research Patient Data Registry database was queried for patients with positive serum MOG-IgG detection, at least once, between January 1, 2017 and March 25, 2021. All were tested via the MOG-IgG1 fluorescence-activated cell sorting assay (Mayo Laboratories, Rochester, MN). MOG-IgG positive cases were reviewed for fulfillment of typical MOG-AD clinical features, determined by treating neurologists and study authors. Of 1,877 patients tested, 78 (4.2%) patients tested positive for MOG-IgG with titer ≥1:20, and of these, 67 had validated MOG-AD yielding a PPV of 85.9%. Using a ≥1:40 titer cutoff, 65 (3.5%) tested positive and PPV was 93.8%. Three MOG positive cases had a prototypical multiple sclerosis diagnosis (RRMS *n* = 2, titers 1:20 and 1:40; PPMS *n* = 1; 1:100). The treating diagnosis for one RRMS patient with a 1:40 titer was subsequently modified to MOG-AD by treating neurologists. Validated diagnoses of the remaining positive patients without MOG-AD included: migraine (*n* = 2, titers 1:20, 1:100), inclusion body myositis (*n* = 1, titer 1:100), autoimmune encephalitis (*n* = 2, titers 1:20, 1:20), hypoxic ischemic brain injury (*n* = 1, titer 1:20), IgG4-related disease (*n* = 1, titer 1:20), and idiopathic hypertrophic pachymeningitis (*n* = 1, titer 1:20). In our cohort, the PPV for MOG-IgG improved utilizing a titer cut-off of ≥1:40. The presence of positive cases with and without demyelinating features, emphasizes a need for testing in the appropriate clinical context, analysis of titer value and clinical interpretation.

## Introduction

Our understanding of the clinical spectrum of myelin oligodendrocyte glycoprotein antibody-associated disease (MOG-AD) is in its infancy when compared to better studied central nervous system demyelinating diseases, such as multiple sclerosis (MS) and neuromyelitis optica spectrum disorder (NMOSD). The advent and further optimization of a live cell-based serum assay for detection of its diagnostic biomarker, MOG-IgG (1, 2), has permitted characterization of the clinical and radiographic spectrum of MOG-AD.

MOG-AD is a CNS demyelinating disorder that affects both pediatric and adult patients with a median age of onset in the fourth or fifth decade of life (3). Clinical manifestations include acute disseminated encephalomyelitis (ADEM), optic neuritis that is often bilateral and/or severe, transverse myelitis that may be short segment or longitudinally extensive, unilateral cortical FLAIR-hyperintense lesions in anti-MOG-associated encephalitis with seizures (FLAMES) (4)/unilateral cerebral cortical encephalitis (UCCE) (5), and brainstem encephalitis (3, 6). Distinct radiographic features of MOG-AD, differing from typical findings in MS, have been described in several studies (7–10). For example, when optic neuritis occurs in the setting of MOG-AD, it is typical to find extensive optic nerve T2 hyperintensity that is chiasm-sparing and with predominant anterior segment involvement on imaging (10).

Its course may be monophasic or relapsing-remitting, and in most cases, the disease is steroid-responsive but may relapse shortly after discontinuing such treatement (3). Currently, MOG-AD is diagnosed when a patient presents with an accepted clinical demyelinating syndrome, has positive detection of MOG-IgG in serum by a validated assay, and does not fulfill criteria for another CNS demyelinating disease (6). Cell-based assays for MOG-IgG detection have been found to have high but imperfect clinical specificity, leading to the potential for false positive results (11, 12). This was highlighted in a study at the Mayo Clinic that found a positive predictive value (PPV) of 72% using a live cell-based assay with a titer cut-off of 1:20 (11).

In this study, we aimed to determine the PPV for a real-world institutional cohort across two academic centers where MOG-IgG ordering is not restricted to neuro-immunologists, but remains available to any physician. We also aimed to discuss indications for serum MOG-IgG testing and utilization of titer cut-offs.

## Methods

The Massachusetts General Brigham Research Patient Data Registry database was queried for patients with positive serum MOG-IgG detection, between January 1, 2017 and March 25, 2021. All were tested via the serum MOG-IgG1 fluorescence-activated cell sorting assay (Mayo Medical Laboratories, Rochester, MN). Identified positive MOG-IgG cases were reviewed for fulfillment of typical MOG-AD clinical features by reviewing authors (GSM, RB). Reviewing authors (GSM, RB) were not blinded to MOG-IgG titer as validation of seropositivity was required for inclusion. Review of the negative serum MOG-IgG cases was beyond the scope of this study. Patients with positive MOG-IgG, clinically consistent MOG-AD, and no more likely alternative diagnosis for their clinical presentation were considered true positives. False positives were patients in whom serum MOG-IgG was detected, but a consistent clinical syndrome was not present or there was felt to be a more likely alternative diagnosis. Positive predictive value was calculated by division of true positives by all positive cases. PPV was further analyzed according to specialty of the ordering provider: neuroimmunologist, neurologist without neuroimmunology expertise and non-neurologist. A Fisher's exact test was used to compare PPV of MOG-IgG when ordered by neuroimmunologists versus neurologists without neuroimmunology expertise.

## Results

### MOG-IgG True Positives and Positive Predictive Value Analysis

During the specified period for data analysis, serum MOG-IgG testing was performed in a total of 1,877 patients, 78 (4.2%) patients were positive. Of these 78 positive patients, 67 patients [39 (58.2%) female; ages 3–81 years (median 28 years)] were identified as true positives using a serum MOG-IgG titer cut-off of ≥1:20. Of all 78 positive MOG-IgG patients with titer ≥1:20, MOG-IgG testing was ordered by neuroimmunologists in 44 (56.4%) cases, by neurologists without neuroimmunology expertise in 33 (42.3%) cases, and by a non-neurologist in 1 (1.3%) case. Initial MOG-IgG testing was predominantly performed within the inpatient setting during acute presentation; for some, initial testing was performed during an outpatient neurology evaluation. A titer cut-off of ≥1:20 yielded a cohort PPV of 85.9% irrespective of ordering provider specialty. The PPV for MOG-IgG ≥1:20 in our cohort when analyzed according to ordering provider specialty was 82% for neuroimmunologists and 94% for neurologists without neuroimmunology expertise (p = 0.17). The single positive test ordered by a non-neurologist was classified as a false-positive. Among the 67 true positive cases, prevalence of clinical phenotypes in order of frequency was: optic neuritis (62.7%, of which 27 cases were unilateral and 15 cases were bilateral), ADEM (14.9%), transverse myelitis (13.4%, of which 5 cases had longitudinally-extensive lesions and 4 cases had short segment lesions), concurrent optic neuritis and transverse myelitis (6%) and brainstem syndrome (3%). Using a MOG-IgG titer cut-off of ≥1:40, PPV, irrespective of ordering provider specialty, improved to 92.3%. Previously published cohort MOG-IgG PPV data is compared with our cohort results in Table 1.

**Table 1:** Comparative table depicting previously published positive predictive values for serum MOG-IgG testing with our cohort PPV.

**Institution**	**MOG-IgG Assay Utilized**	**Cohort PPV** **(MOG-IgG Titer Cut-off)**
Mayo Clinic (11)	Mayo Clinic cell-based FACS MOG-IgG1 assay	72%, irrespective of titer 51% (1:20–1:40) 82% (1:100) 100% (1:1,000)
MGH/BWH Cohort	Mayo Clinic cell-based FACS MOG-IgG1 assay	85.9% (≥1:20) 92.3% (≥1:40)

*FACS Fluorescence-activated cell sorting; IF immunofluorescence; NR not reported*.

### MOG-IgG False Positives

Among the total 1,877 patients tested during this study period, 11 cases were deemed false positives. False positives were MOG-IgG seropositive with titer of ≥1:20, but had a clinical syndrome not consistent with MOG-AD and/or a more likely alternative diagnosis. The clinical diagnoses of the false positive cases included both non-inflammatory and inflammatory neurologic diseases as detailed in Table 2. The specialty of ordering provider for each false positive case is also included in Table 2. Rationale for MOG-IgG testing in these cases was documented by the clinical care team. In the case of inclusion body myositis, MOG-IgG was tested due to an initial concern for a T-cell-mediated inflammatory myopathy with concurrent nonspecific white matter lesions noted on brain MRI. MOG-IgG was tested in the patient with known IgG4-related renal disease due to new vision loss secondary to acute optic neuritis and perineuritis. In the two cases later diagnosed as autoimmune encephalitis, MOG-IgG testing was initially prompted by abnormalities noted on brain MRI.

**Table 2:** Clinical diagnoses and corresponding serum MOG-IgG titers of false positive cases.

**Clinical Diagnosis, *n***	**Serum MOG-IgG Titer**	**MOG-IgG ordering provider specialty**
Autoimmune Encephalitis (AE), *n* = 2 - AE with cerebellar degeneration - Post-herpes simplex virus NMDAR AE	1:20 1:20	Neuroimmunologist Neurologist
Hypoxic ischemic brain injury, *n* = 1	1:20	Non-neurologist
Idiopathic hypertrophic pachymeningitis, *n* = 1	1:20	Neuroimmunologist
IgG4-related disease, *n* = 1	1:20	Neurologist
Inclusion body myositis, *n* = 1	1:100	Neuroimmunologist
Migraine with aura, *n* = 2	1:20 1:100	Neuroimmunologist Neuroimmunologist
Primary Progressive MS, *n* = 1	1:100	Neuroimmunologist
Relapsing Remitting MS, *n* = 2	1:20 1:40	Neuroimmunologist Neuroimmunologist

## Discussion

Optimization of the live cell-based MOG-IgG assay has provided a reliable, diagnostic tool with robust specificity, permitting MOG-AD characterization (1, 2). In practice, validation of the clinical applicability of such a diagnostic test within a given population can be demonstrated via positive predictive values; such has been published for the MOG-IgG live cell-based assay previously (11, 12), and our study further supplements this aim. The fact that what is defined as MOG-AD is based upon currently accepted clinical phenotypes, without a more objective gold standard, is a general limitation of this study. An additional limitation pertains to the definition of a false positive MOG-IgG result. It is possible that overlap neuro-inflammatory syndromes may exist; however, in our cohort some neuroinflammatory cases were classified as false positives due to a more likely alternative diagnosis despite MOG-IgG seropositivity. Other study-specific limitations include the retrospective design and lack of repeated testing for most patients.

A positive correlation between serum MOG-IgG titer cut-off value and PPV for MOG-IgG has previously been reported (11). Sechi et al. (11) has published titer dependent PPV: PPV 100% for 1:1000, 82% for 1:100, 51% for titers 1:20–1:40 (Table 1) (11). A titer threshold greater than or equal to 1:40, rather than greater than or equal to 1:20, similarly improved the PPV for MOG-IgG in our cohort from 85.9 to 92.3%; yet, excluding patients with titers of 1:20 without proper clinical analysis may lead to clinical inadequacy (13). In our study, seven cases with MOG-IgG titers of 1:20 were found to have clinical MOG-AD, equating to a true positive status using the initial cut-off of greater than or equal to 1:20. This exemplifies that numerical cut-off values, although important for assay specificity and reliability at the population level, will continue to require supplementary clinical interpretation. Similarly, determination of false positive status equally warrants appropriate clinical interpretation. Reassuringly, MOG-IgG ordering by neurologists with and without neuroimmunology expertise separately yielded robust PPV for MOG-IgG in this cohort. The PPV of MOG-IgG when ordered by a non-neurologist cannot be determined from our study, as MOG-IgG was ordered by a non-neurologist in only one instance. It is important to acknowledge that PPV is dependent upon disease prevalence within a given population. Thus, our findings can be best applied to similar patient cohorts in which testing is pursued for patients presenting with congruent neurologic symptoms.

The determination of false positives prompt consideration as to whether conservative MOG-IgG testing permits a full understanding of MOG-AD. Among the eleven false positive cases in our cohort, three had CNS demyelinating disease, albeit multiple sclerosis, and five patients had other neuroimmunologic conditions (Table 2). However, three patients had non-neuroimmunologic conditions, and low levels of MOG-IgG have been detected in patients with diverse neuroimmunologic and non-neuroimmunologic diagnoses previously (14). The clinical relevance of MOG-IgG positivity in patients with atypical phenotypes for MOG-AD and/or more likely alternative diagnoses, such as multiple sclerosis, is thus an area that would benefit from future exploration (13, 15).

In what is presently defined as “atypical” for MOG-AD, should MOG-IgG be tested? Do testing restrictions limit full appreciation of the MOG-AD spectrum? Ongoing investigations are needed to better solidify the spectrum of MOG-AD phenotypes, differentiating atypical presentations as being either part of or outside of the spectrum of disease with false bystander MOG-IgG positivity.

## Data Availability Statement

The raw data supporting the conclusions of this article will be made available by the authors, without undue reservation.

## Ethics Statement

The studies involving human participants were reviewed and approved by Mass General Brigham IRB. Written informed consent for participation was not required for this study in accordance with the national legislation and the institutional requirements.

## Author Contributions

GM contributed to data acquisition and analysis, manuscript drafting and editing, and table creation. RS contributed to data acquisition and manuscript editing. FM, EK, and TC contributed to critical review and commentary or revision for the manuscript. ML and MM contributed to data analysis, critical review, and commentary or revision for the manuscript. All authors contributed to the article and approved the submitted version.

## Conflict of Interest

ML has received consulting fees from Sanofi, UCB and Genentech. The remaining authors declare that the research was conducted in the absence of any commercial or financial relationships that could be construed as a potential conflict of interest. The handling editor GF declared a past co-authorship with the author TC.

## Publisher's Note

All claims expressed in this article are solely those of the authors and do not necessarily represent those of their affiliated organizations, or those of the publisher, the editors and the reviewers. Any product that may be evaluated in this article, or claim that may be made by its manufacturer, is not guaranteed or endorsed by the publisher.
